# Routes for Specialization in Psychology throughout Europe

**DOI:** 10.3390/bs10010007

**Published:** 2019-12-19

**Authors:** David Dias Neto, Sónia Figueiredo, Constança Biscaia, Maria João Barros, Ricardo Barroso, Filipa Fernandes

**Affiliations:** 1 APPsyCI—Applied Psychology Research Center Capabilities & Inclusion, ISPA—Instituto Universitário, Lisboa 1100-304, Portugal; 2 Ordem dos Psicólogos Portugueses, Lisboa 1050-116, Portugal; sonia.figueiredo@ordemdospsiscologos.pt (S.F.); cbiscaia@uevora.pt (C.B.); maria.barros@ordemdospsicologos.pt (M.J.B.); rbarroso@utad.pt (R.B.); 3 LabCom.IFP-Comunicação, Filosofia e Humanidades, Universidade de Évora, Évora 7000, Portugal; 4 Department of Education and Psychology, University of Trás-os-Montes and Alto Douro, Vila Real 5000-801, Portugal; 5 Clinical and Health Psychology Department, ISPA—Instituto Universitário, Lisboa 1100-304, Portugal; analipa21@gmail.com

**Keywords:** specialties of psychology, EuroPsy, training

## Abstract

The specialization of psychology helps to delineate fields in the practice of psychology. When establishing professional qualification criteria, associations seek to promote, in their members, scientific skills and knowledge considered fundamental for the practice of psychology in a given area. The present study reports on a survey of the member associations of the European Federation of Psychologists Associations (EFPA). The survey inquired about: (a) the initial requisites for entering the profession and (b) additional requisites for specialization. Of the 37 associations contacted, 14 replied and we retrieved the information of 12 associations from their official websites. The results indicate that specialization is widespread throughout Europe and is independent of the regulation of the profession. In almost half of the countries considered, the specialization process is completed in universities—it is frequently associated with the postgraduate level of the EuroPsy—and relies on conventional learning methods. The number of existing specialties in psychology is very high, but the traditional areas (clinical and health, education, and social/organizational) are more prevalent. The results are discussed in light of the advantages, but also the challenges posed by the specialization in psychology. A continuous model of the specialization of psychology is proposed with two stages: broad and advanced psychology areas.

## 1. Introduction

The idea of specialization as a constitutive professional element is entrenched in European culture. It is influenced by the works of Adam Smith and Charles Darwin [1], in which it is seen as a way for improving productivity and the quality of work and as a means by which a species adapts to their environment. Specialization is also consonant with the rapid progression of science in general, of which psychology is no exception. The perceived durability of knowledge is, on average, 8.68 year for different fields of psychology [2]. In professional organizations, specialization processes and specialization titles constitute hallmarks to promote professional development. In these organizations, specialization frameworks often result from professional consensus and reflect different professional realities.

It is important to realize that even psychology, with its relatively short history as a science, was born as a specialization of other fields. Currently, psychology, as a science, is well defined, but there is still room for development at a professional level. There has been significant effort to define training standards to practice psychology, based on a broad consensus effort [3,4,5,6,7]. Presently, in Europe, psychology is reasonably regulated around similar standards. Even where it is not regulated by national law, there are regional or specific standards that regulate the activity of psychologists [8]. One important contribution to this normalization is the establishment of training criteria—the EuroPsy—by the European Federation of Psychologists’ Associations (EFPA). 

The EuroPsy standard [9], proposed for the practice of psychology, requires candidates to complete five years of university education with a recognized curriculum, plus one year of supervised practice. In countries that have gone through the Bologna process, the five years are typically done in a 3-year graduate level (e.g., BA or BSc) and 2-year postgraduate level (e.g., MA or MSc). The EuroPsy also implies abiding by the code of ethics and undertaking continuing professional development.

The criteria for further specialization is significantly less defined. Currently, EFPA has two specialist certificates [10] for psychotherapy and work and organizational psychology. However, only a few countries can apply to these certificates and the fact that they have different training requirements suggests that there is no broad framework for specialization underlying these specialties. They do, however, show that after the generalization of the EuroPsy, the specialist EuroPsy will gain relevance. This is supported by the observation that frameworks for specialization already exist at a national level.

The need for specialties in psychology is a reflection of the perceived advantages of specialization [5,11]. Specialties provide a means for professional enrichment, valorization of training, delineation of specific contexts of practice, and can be a process for rewarding merit and competency. At a European level, if this specialization is recognized, it can allow for mobility and sharing of knowledge between nations.

There are, however, important disadvantages of specialization. It can lead to an artificial division of psychologists and psychology. It can constrict the labor market by creating restrictive professional categories and can favor specific models of psychology and be the result of confusion between scientific and professional areas. Finally, the idea of being a specialist may reduce the motivation for continuous professional development. Psychologists may believe that achieving a specialization is the endpoint of professional development. Furthermore, in less developed countries or regions with low population density, despecialization may be a necessity, and some lessons can be drawn from these contexts [11]. A more generic approach to the practice of psychology may reveal that some problems are better addressed in an integrated way. For example, a clinical problem may also be addressed with a social or community psychology approach. Excessive compartmentalization may overlook potential solutions to societal challenges.

To inform the discussion on the specialization in psychology, it is crucial to understand the current situation. To present a picture of the current state of specialization in psychology in Europe, we conducted a short survey directed at member associations of EFPA. This study has the following goals: (a) to assess if member associations have formal frameworks for specialization in different areas of psychology, and (b) identify the areas of specialization chosen by each member association. It is important to consider that there is still significant diversity in the regulation for “general” psychology. Therefore, for example, for some countries, specialization may refer to the second or postgraduate level of the EuroPsy (3 + 2 years), while for others, it is a training done after becoming a psychologist (3 + 2 + x years). Furthermore, member associations of EFPA differ significantly in their regulatory role of psychologists. Some countries mandate the member association to regulate some aspects of all psychologists, which imply the mandatory registration of all psychologists in that association, while others are professional associations whose members adhere voluntarily. This has several consequences, particularly in the meaning and legal role assigned to the specialist title.

## 2. Materials and Methods

### 2.1. Participants

Member associations of EFPA were contacted by email and asked to complete a small survey. To facilitate response, member associations could reply via a Google form or by directly replying to the email. Data collection occurred between 26 March, 2019 and 26 April, 2019. When the member associations did not reply, the information on the survey was sought on the official website of the association. When the information was not explicit, we opted for not including it. Both the emails and the websites were retrieved from the EFPA webpage.

### 2.2. Materials

Table 1 presents the questions of the survey sent to the EFPA member associations. The survey was divided into three parts. In the first part (Question 1), the goal was to inquire about the general requirements for becoming a psychologist in that particular country. This is important because this baseline is important to interpret the following questions. The second part referred to the countries’ framework for specialization in psychology (Questions 1–5). This section asked for the specialty areas considered in each country, the training requisites, and characteristics. The final section (Question 6) referred to other forms of delineation of specialized areas in psychology.

## 3. Results

Of the 37 members of EFPA contacted, 14 associations replied to our email, and we retrieved some information from an additional 12. The raw information is provided in Appendix A to allow for a more detailed country comparison. 

### 3.1. Current State of Specialization in Psychology

Of the 37 associations of the EFPA, 17 had formal procedures for recognizing specialties in psychology, and only two reported not having such procedures. Table 2 presents the distribution of the 17 countries considering the level of initial training (i.e., for being recognized as a psychologist). The results suggest a similar distribution to the total number of associations. A total of 75.0% of the countries with a framework for specialization had at least five years of training as requisite for being a psychologist (C + D), which is close to the 73.9% found in all associations. This suggests that there is no relationship between the level of requisites for being a psychologist and the existence of specialization.

**Figure 1:**
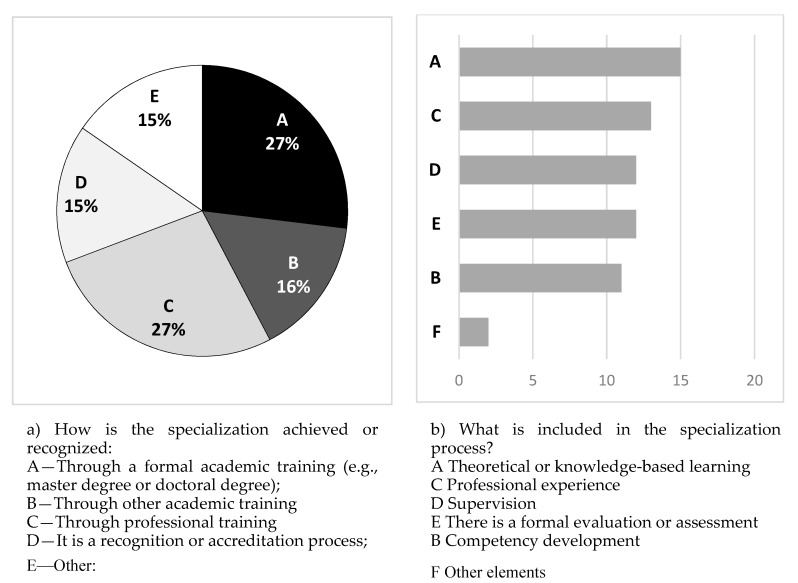
Type and components of training required for specialization.

The results of the procedures for becoming as a specialist and the contents of the specialization process are presented in Figure 1. A total of 43% of the specialization in psychology was conducted in universities (Options A and B). This is explained by the fact that in a relevant number of countries, the specialization corresponds to the second or postgraduate (e.g., MA, MSc) level of training in psychology. In countries where the specialization occurs after these two levels, the training is typically a professional recognition or accreditation procedure or specific training. With respect to the components of the specialization process, the most frequent components are theoretical training, professional experience (e.g., practicum), and supervision. Other elements or a specific focus on competency development are less frequent.

### 3.2. Areas of Specialization in Psychology

Appendix A shows a significant diversity in the areas of specialty considered by the EFPA’s associations with a high number of specialties specific to a particular country. The ten most common areas are:

1. Social, organizations, and work psychology (13)
2. Clinical psychology or clinical and health psychology (12)
3. Child and adolescent psychology or child and family psychology (9)
4. Neuropsychology or clinical neuropsychology (9)
5. Educational or school psychology (8)
6. Psychotherapy (8)
7. Health psychology (5)
8. Forensic psychology or psychology of justice (4)
9. Counseling (4)
10. Gerontopsychology (3)
11. Sports psychology (3)

The same distribution was found when interest areas were considered. The areas recognized in more than one country were: social, organizations, and work psychology (25); clinical psychology or clinical and health psychology (20); educational or school psychology (19); child and adolescent psychology or child and family psychology (15); psychotherapy (13); neuropsychology or clinical neuropsychology (12); health psychology (12); forensic psychology or psychology of justice (12); sports psychology (11); gerontopsychology (8); traffic or transport psychology (8); emergency or crisis psychology (7); counseling (4); military psychologist (4); trauma psychology or psychotraumatology (4); sexology or psychology of sexuality (3); testing in psychology or differential psychology (3); addiction psychology (3); ecological psychology (2); history of psychology (2); mediation (2); special educational needs (2); community psychology (2), and coaching psychology (2).

Several observations can be drawn from this list. First, the sheer diversity. Most specialties, shared by two or more countries were, nevertheless, present only in a few countries. The exceptions are traditional areas: clinical and health psychology, education psychology, and work and organizational psychology. Clinical psychology is often divided into adult and child/adolescent interventions. Finally, some areas like psychotherapy were considered, probably as a way to affirm these practices when conducted by psychologists.

## 4. Discussion

The picture of psychology specialization shows that it is still in its infancy. Nevertheless, specialization in different psychological areas is very common in Europe. Only two countries stated not having a specialization process, but even in one of these, there are informal interest groups in specialized areas. Given this popularity, it is important to situate the interpretation of the results in consideration of the advantages and disadvantages of specialization. This is particularly relevant with respect to two issues: (a) the lack of delineation of the difference between a psychologist and a specialist and (b) the overlap between specialized areas.

The results of our survey suggest that there are different means and timings for acquiring a specialty in psychology. In some countries, by completing the 3 + 2 years of university education and a year of professional experience, the title acquired is that of psychologist. Specialized training is initiated afterward. In other countries, the second or postgraduate level of training in psychology is a synonym of a specialization. This has two important implications. First, it implies different levels of demand throughout Europe. Second, and more importantly, it weakens the profession of psychology. If the notion of a general psychologist (or simply of psychologists) is eliminated, then psychology is a federation of professions. Furthermore, given the overlap between specialized areas, the affirmation of some areas is achieved at the cost of others.

The model of specialization should take into account both the unity of psychology and the differentiation in specific areas. This means that the specialties should be thought of in an integrated way, rather than as a response to particular interest groups. We have previously proposed [12] a few principles that could guide the development of a specialization framework. Independently of the particular model each country chooses, it is important to maintain the unity of psychology while respecting professional and scientific diversity. 

The second problem identified in the survey is the overlap between specialized areas. The number of specialized areas considered was substantial. This diversity suggests that the fields of specialization are not consensual and not as established as in other professions. This supports the concerns that specialization may promote excessive polarization of psychological practice. On one hand, the different proposals of areas of psychology reflect the diversity in psychology. On the other hand, the vast diversity of proposed specialties threatens to fragment psychology. The only consensus that can be observed throughout Europe is on the three traditional areas of psychology: clinical and health psychology, education psychology, and work and organizational psychology. However, the existence of the other areas implies that specific areas within psychology should be acknowledged.

One way to deal with this issue is by thinking of the specialization process in an integrated way, but also in a more continuous way. The EuroPsy recognizes three areas of practice: clinical and health psychology, work and organizational psychology; and educational psychology. These correspond to the traditional areas identified throughout Europe. One possible way to think of the relation between these broader areas and the remaining more specific ones is to consider them as two levels of specialization [13]. 

**Figure 2:**
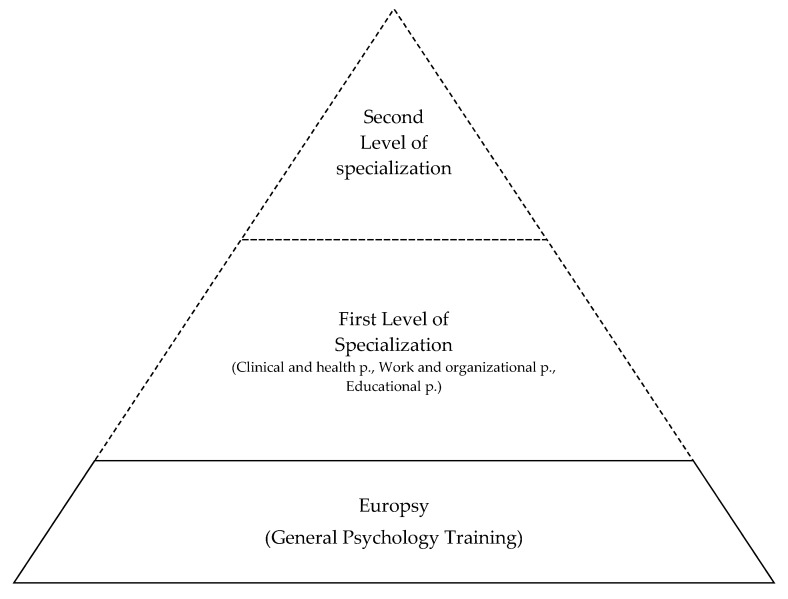
Continuous specialization in psychology.

The model that we are arguing for (see Figure 2) would imply understanding specialization as a progressive endeavor. After completing the general training, psychologists could be considered simply as psychologists. Specialization could then be initiated in one of the three general areas of psychology with progressive specialization—when desired—into areas that are more specific. It is important to note that the three general areas are those consensually considered today. In the future, other areas could be considered as broad areas in the function of scientific or professional progress. A continuous model of specialization attenuates the potential for polarization or rigid boundaries. 

This survey had several limitations. First, the number of replies was small, and there was no control over the answers provided by the member associations. What can be considered as a formal framework for specialization can be different in different countries. As above-mentioned, this confusion extends to what is considered as a psychologist. This limitation is a direct consequence of the different terms used throughout Europe on professional issues. Future research using other methods such as focus groups may circumvent these language and conceptual barriers.

Despite these considerations, this survey suggests that specialization in psychology is inevitable. It is important to reflect on these processes, taking into account their impact on psychology as a profession or as science. Metaphorically, specialization can be seen as a centrifugal force that can be important in the development of psychology. It is important that this force is balanced to preserve the unity of psychology and the strength of its practice. 

## Figures and Tables

**Table 1:** Survey.

Question
**1. What are the requisites for practice Psychology (as a non-specialist) in your country?** A—Our country has no requisites; B—Bachelor degree, or equivalent, in psychology (3 years); C—Bachelor and master degree, or equivalent, in psychology (3+2 years); D—3 + 2 years of academic training in psychology + 1 year of professional experience or practicum (EuroPsy); E—Other
**2. Does your country have a national framework for the specialization in psychology?** (By specialization framework we mean different of rulings (e.g., training, professional development, practice criteria) that allow a psychologist to use a specialized title or be recognized, nationwide, as having a specialization in psychology) A: Yes; B: No. Please select “No” if your country only has scientific or professional interest-areas that are self-acknowledged by psychologists (If not, please move to Question 6)
**3. What areas of specialization (e.g., social psychology, educational psychology) are there in your country?**
**4 How is the specialization achieved or recognized?** A—By means of formal academic training (e.g., master degree or doctoral degree); B—By means of other academic training (that may include professional experience or practicum); C—By means of professional training (that may include professional experience or practicum); D—It is a professional/training recognition of accreditation process; E—Other:
**5 What is included in the specialization process (in either the training or the recognition process)? Please select as many as required.** A—Theoretical or knowledge-based learning; B—Competency development (implies a specific formulation of the required competencies); C—Professional Experience; D—Supervision; E—There is a formal evaluation or assessment; F—Other elements:
**6. Other than the recognized national specialties of psychology, does your association have permanent psychology interest-areas or groups? If so, in what areas of psychology (e.g., educational psychology, clinical psychology, sports psychology, traffic psychology)?**

**Table 2:** Existing frameworks for specialization according to the level of requisites for practicing psychology.

Requisites for Psychology	With a Framework for Specialization	Total
A—Our country has no requisites	2 (12.5 %)	5 (21.7 %)
B—BA degree, or equivalent (3 years)	0 (0 %)	0 (0 %)
C—BA and MA or equivalent (3 + 2 years)	10 (62.5 %)	12 (52.2 %)
D—EuroPsy	2 (12.5 %)	5 (21.7 %)
E—Other	2 (12.5 %)	1 (4.3 %)
Unknown (-)	1	14
Total	17	37

## Appendix A

### Appendix A.1. Answers to the Survey or Information Retrieved from the Official Website

Questions: (1). What are the requisites for the practice of psychology (as a non-specialist) in your country? (2) Does your country have a national framework for specialization in psychology? (By specialization framework we mean different of rulings (e.g., training, professional development, practice criteria) that allow a psychologist to use a specialized title or be recognized, nationwide, as having a specialization in psychology). (3) What areas of specialization (e.g., social psychology, educational psychology) are there in your country? (4) How is the specialization achieved or recognized? (5) What is included in the specialization process (in either the training or the recognition process)? Please select as many as required. (6) Other than the recognized national specialties of psychology, does your association have permanent psychology interest-areas or groups?

**Source**	**Country**	**1**	**2**	**3**	**4**	**5**	**6**
Survey	**Austria—**Berufsverband Österreichischer Psychologinnen	C	Yes	- Gerontopsy. child, youth and family psy.; clinical neuropsy.; emergency psy.	C	A, B, C	Work, economic and organizational psy.; mediation; educational psy.; forensic psy.; sports psy.; trauma psy.; traffic psy.; addiction psy.
Website	**Belgium—**Belgische Federatie Van Psychologen	C	-	-	-	-	-
Survey	**Bulgaria—**Bulgarian Psychological Society	E	Yes	General psychologist; clinical psychologist; school psychologist; psychotherapist.	A or B or C or D	A or B or C or D or E	Planned: child psychologist; school psychologist; clinical psychologist; psychotherapist; health psychologist; advisory psychologist; crisis psychologist; social and political psychologist; labor and organizational psychologist; Legal psychologist; military psychologist; Transport psychologist; sports psychologist.
Website	**Croatia—**Hrvatsko Psihologijsko Društvo	-	-	-	-	-	Forensic psy.; clinical psy.; psy.in occupational medicine; psychological psy.; development psy. for early and preschool age; psy. of developmental disabilities and disabilities; psy. of work and organizational psy.; psy.in social welfare; sports psy. and physical exercise; school psy.; military psy.; psy. in palliative care; psy. of sexuality and psy. of gender; traffic psy.; family psy.; marriage and partnerships; aging psy.; health psy.; psychodynamic psy.; ecological psy.; human rights and psy.; history of psy.
Survey	**Cyprus—**Cyprus Psychologists' Association	C	*Yes*	Clinical psy.; counselling psy.; forensic psy.; school/applied educational psy.; Industrial/organization psy.	A, B, C, D, E	A, B, C, D, E	-
Website	**Czech Republic**—Unie Psychologických Asociací	C	-	-	-	-	-
Website	**Denmark**—Dansk Psykolog Forening	C	Yes	Child psy. (clinical child psy., clinical child psy., pedagogical psy., health psy. with children and psychotherapy with children); adult psy. (health psy. with adults, psychotherapy with adults, psychopathology, psychotraumatology, clinical neuropsy., gerontopsy.); work and organizational psy.	C	A, B, C, D, E	-
Survey	**Estonia—**Eesti Psühholoogide Liit	A	Yes	Clinical psy.; school psy..	A	A, C, D, E	No
Survey	**Finland**—Suomen Psykologiliitto	C	Yes	Child and adolescent psy.; work and organizational psy.; health psy.; neuropsy.; psychotherapy; adult clinical psy.	B	A, B, C, D, E	-
Website	**France—**Fédération Française Des Psychologues Et De Psychologie	A	-	-	-	-	-
Website	**Germany—**Föderation Deutscher Psychologenvereinigungen	C	-	-	-	-	General psy.; work, organizational, and economic psy.; biological psy. and neuropsy.; differential psy., personality psy. and psychological diagnostics; developmental psy.; history of psy.; health psy.; clinical psy. and psychotherapy; media psy.; methods and evaluation; educational psy.; forensic psy.; social psy.; sports psy.; ecopsy.; traffic psy.
Website	**Hungary—**Magyar Pszichológiai Társaság	C	Yes	Clinical and/or health psy.; cognitive psy.; counseling and/or school psy.; social and organizational psy., work and organizational psy.; developmental and clinical psy. of children; intercultural and interpersonal psy.	A	A, B, C, D, E	-
Website	**Italy—**Associazione Unitaria Psicologi Italiani	D	-	-	-	-	-
Website	**Liechtenstein**—Berufsverband Der Psychologinnen Und Psychologen Liechtensteins	A	-	-	-	-	Psychotherapy; neuropsy.; traffic psy.; health psy.; school psy.; gerontopsy.; forensic psy.; parenting/family/education; job/career/work
Survey	**Lithuania—**Lietuvos Psichologų Sąjunga	C	Yes	School psychologist; medicine psychologist	E	A	Educational psy.; clinical and health psy.; crisis and catastrophe psy.; organizational psy.; law psy.
Survey	**Luxemburg—**Société Luxembourgeoise De Psychologie	A	No	-	-	-	Educational psy.; testing in psy.; psychotherapy; neuropsy.; psy. in state child care settings.
Survey	**Malta—**Malta Chamber Of Psychologists	C	Yes	Clinical, counselling, health, forensic, educational, social, sports	A	A, B, C, D	-
Survey	**Netherlands—**Nederlands Instituut Van Psychologen	A	Yes	Mental health care; clinical (neuro)psychologist; child psychologist; psychotherapist	A, C, E.	A, C, D, E	Work and organizational psy.; forensic psy.; Body-oriented working psychologist; mediator; neurofeedback psychologist; psychologist in somatic healthcare; Revalidation; sexuality and diversity; social and economic psy.; addiction psy.; care for people with intellectual disabilities.
Survey	**Norway**—Norsk Psykologforening	E	Yes	Adult psy.; children and youth psy.; elderly psy.; community psy.; neuropsy.; family psy.; addiction psy.; habilitation psy.; organizational psy.; work psy.; psychotherapy.	B, C, D	A, B, C, D, E	Neuro psy.; forensic psy.; private practice; intercultural psy.; digital health psy.; addiction psy.
Survey	**Portugal—**Ordem dos Psicólogos Portugueses	D	Yes	Social, work and organizations psy. (psychological coaching; community psy.; occupational health psy.); educational psy. (early intervention; vocational and career development psy.; special educational needs); clinical and health psy. (neuropsy.; psycho-gerontology; psy. of justice; psy. of sport; psychotherapy; sexology)	D	A, B, C, D, E, F	-
Website	**Romania—**Colegiul Psihologilor din România	C	-	-	-	-	Clinical psy. and psychotherapy; labor transport psy.; educational psy.; school counseling and vocational counseling; psy. for public order defense; and national security.
Survey	**San Marino—**Ordine degli Psicologi della Rep. di San Marino	D	No	-	-	-	-
Website	**Serbia—**Društvo psihologa Srbije	-	-	-	-	-	Educational psy.; organizational and work psy.; clinical psy.
Website	**Slovakia—**Slovenska Komora Psychologov	C	Yes	Clinical psy.; counseling; organizational and work psy.	A	-	-
Survey	**Slovenia**—Drustvo Psihologov Slovenije	C	Yes	Clinical psy.	C	A, B, C, D, E	Sport psy.; work and organizational psy.; psy. in social welfare; educational psy.; psychotherapy; health psy.; c. for psychodiagnostical tools, c. for ethics, c. for forensic psy. of family.
Website	**Spain—**Consejo General de Colegios Oficiales de Psicologos	-	Yes	clinical psychology; neuropsy.; palliative care psy.; sport psy.; aeronautical psy.; emergency and disaster psy.; educational psy.; social Intervention psy.	-	-	-
Website	**Sweden—**Sveriges Psykologförbund	D	Yes	organizational and work psy.; educational psy.; psychological treatment/psychotherapy; neuropsy.; clinical adult psy.; clinical child and adolescent psy.; health psy.; addiction’s psy.; disability's psy.; forensic psy.	-	A, B, C, D, E	-
Survey	**Switzerland—**Föderation der Schweizer Psychologen/innen	C	Yes	Psychotherapy; child and youth psy.; clinical psy.; neuropsy.; health psy.	E	A, E, F	Coaching psy.; career and personnel psy.; legal psy.; sports psy.;traffic psy.; emergency psy.; psychooncology; gerontopsy.; cognitive behavioral therapeutic supervision; psychotraumatology.
Website	**Turkey—**Türk Psikologlar Derneği	-	-	-	-	-	Trauma, disaster and crisis unit, department of child and adolescent studies, test unit, department of traffic psy., publication unit, LGBTI studies unit, clinical psy. unit, health psy. unit, sports psy. unit, women and gender studies unit.

## References

[1] Moghaddam B.M. (2009). The psychology of specialization and specialization in psychology. Eur. J. Psychol..

[2] Neimeyer G.J., Taylor J.M., Rozensky R.H., Cox D.R. (2014). The diminishing durability of knowledge in professional psychology: A second look at specializations. Prof. Psychol. Res. Pract..

[3] Peiro J.M., Lunt I. (2002). The context for an European framework for psychologists’ training. Eur. Psychol..

[4] Donn J.E., Routh D.K., Lunt I. (2000). From Leipzig to Luxembourg (via Boulder and Vail): A history of clinical psychology training in Europe and the United States. Prof. Psychol. Res. Pract..

[5] Hall J.E. (2005). Global mobility for psychologists: The role of psychology organizations in the United States, Canada, Europe, and other regions. Am. Psychol..

[6] Lunt I. (2002). A common framework for the training of psychologists in Europe. Eur. Psychol..

[7] Lunt I. (1999). European framework for psychologists training: Project funded by the European Union under the Leonardo program. Eur. Psychol..

[8] Van Broeck N., Lietaer G. (2008). Psychology and psychotherapy in health care: A review of legal regulations in 17 European countries. Eur. Psychol..

[9] European Federation of Psychologists’ Associations EuroPsy. https://www.europsy.eu/quality-and-standards/europsy-basic.

[10] European Federation of Psychologists’ Associations Specialist EuroPsy. https://www.europsy.eu/quality-and-standards/europsy-specialisation.

[11] Moghaddam F.M. (1989). Specialization and despecialization in psychology: Divergent processes in the three worlds. Int. J. Psychol..

[12] Neto D.D. (2014). Sete princípios orientadores das especialidades. Psis21.

[13] Drum D.J., Blom B.E. (2001). The dynamics of specialization in professional psychology. Prof. Psychol. Res. Pract..

